# National Medical Convention

**Published:** 1846-04

**Authors:** 


NATIONAL MEDICAL CONVENTION.

DR. J. V. Z. BLANEY:

Dear Sir,—In obedience to the last resolution appended to the accompanying report, I send it to you, hoping that you will give it an insertion in the April No. of the "Illinois Medical & Surgical Journal."

It is now certain that a full Convention will be held, and we hope the Medical College at Chicago, as well as the State of Illinois, will be fully represented therein.

Very respectfully, yours, &c.,
N. S. DAVIS,
Binghamton, Broome Co., N. Y.

---

The Committee appointed at the last Annual Meeting of the New York State Medical Society, to carry into effect a resolution passed at the same meeting, inviting a National Medical Convention in May next, would respectfully report as follows:

In the early part of last season, the Committee through their Chairman, addressed a circular, containing the preamble and resolutions of this Society, with such comments as were deemed advisable, to all the State Medical Societies and Colleges in the United States, as far as the existence of such Societies and Colleges could be ascertained. And in those States where neither State Societies nor Colleges existed, a circular was addressed to some leading member of the profession, inviting him to take measures for having his State represented in the proposed Convention. Replies to these circulars and letters have been received from the following officers of Medical Societies and Colleges, and private members of the profession, viz: Drs. W. W. Morris, of Dover, Delaware State; A. H. Buchanan, of Tennessee; W. P. Johnston, of Washington City; T. T. Hewson, R. M. Huston, and W. E. Horner, of Philadelphia; Luther Ticknor, of Connecticut; W. H. McKee, of North Carolina; E. H. Pearlee, of Hanover; Paul F. Eve, of Georgia; J. H. Thompson, of New Jersey; J. W. Davis, of Indiana; A. Twitchell, of New Hampshire; J. W. Draper, A. H. Stephens, Willard Parker, and C. A. Lee, of New York; J. Drake, of Ohio; L. M. Lawson of Kentucky; and Professor Carpenter of Louisiana. And delegates have been freely pledged from Societies and Colleges in Maine, New Hampshire, Connecticut, New Jersey, Delaware, District of Columbia, South Carolina, Georgia, Mississippi, Louisiana, Kentucky, Ohio, Indiana, and New York. The medical schools of Philadelphia are the only ones from whom replies have been received, that decline sending delegates and giving a hearty support to the proposed measure. Nearly every medical journal throughout the Union, has also, not only favorably noticed, but warmly commended the proposition for holding such a convention. There are some Institutions from whom no replies have been received, but from information obtained from other sources, there is good reason for believing that most, if not all these will be represented by the appoitment of delegates, as soon as they are fully assured that the convention *will be* held. It will thus be seen that in far the larger part of the Union, the invitation of the society has met with a prompt and hearty response from the profession; and it is with much regret that we find even a few Institutions declining to to take any part in so important a movement. But when we consider the wide extent of our territory, and the great number of our Institutions, all engaged, we should hope, in a generous rivalry with each other, the expression in favor of a convention, is certainly more unanimous and more promising of good than could have been anticipated. Indeed the leading and influential members of the profession have long felt the necessity of some national action, some central point of influence, around which the active and choice spirits of the whole profession can rally; from which may be made to radiate an elevating, healthful, and *nationalising* influence over the whole country.

Hence it only remains for this Society to carry out the work it has so nobly commenced. The Faculty in the New York University have very generously tendered to this society the use of any room or rooms in their College edifice, that may be desired for the Convention to meet in. Some State Societies and Colleges have already appointed their delegates, while others have expressed a desire that this Society would fix the number to be sent from any one Society or College.

For the purpose of furthering the objects in view, your Committee would respectfully submit the following resolutions, viz:

1. *Resolved*, That the preamble and resolutions, passed by this Society at its annual session Feb. 6, 1845, did not contemplate the appointment of delegates to the National Convention, from county or merely local societies, in those States where delegates *are* appointed by a regularly organized *State* Society.

2. *Resolved*, That as some Societies and Colleges have already appointed their delegates, therefore the number to be appointed by any one of these Institutions should be left entirely to the discretion of the appointing body.

3. *Resolved*, That sixteen delegates be appointed to represent this Society in the proposed National Convention, viz: two from each senatorial district in the State.

4. *Resolved*, That this Society accept the offer of the Faculty in the New York University respecting their *rooms;* and consequently that the members of said National Convention are invited to assemble in the College edifice of the New York University, at 10 o'clock, A. M., on the *first Tuesday* in May next.

5. *Resolved*, That a Committee of three be appointed to continue the efforts to further the objects of the proposed Convention, and to exert all their influence to make it truly *National* in *composition* and *action.*

6. *Resolved*, That the above Committee be authorized to invite the Superintendants of Lunatic Asylums throughout the several States to attend the Convention.

7. *Resolved*, That the Committee be further authorised and enjoined to send a copy of this report, together with the accompanying resolutions, and the action of this society thereon, to all the medical journals in the United States, with a request that they publish the same on or before the first of April next.

N. S. DAVIS,<br>

JAMES McNAUGHTON, } *Committee.*<br>

PETER VAN BUREN,

Albany, Feb. 3, 1846.

The foregoing report was accepted, and the resolutions unanimously adopted. Sixteen delegates were appointed according to the third resolution; and the above named Committee re-appointed in obedience to resolution fifth.

N. S. DAVIS, *Ch'n. Com.*

We lay before our readers the foregoing report of the committee of the New York State Medical Society, with much pleasure; nor are we at all surprised at the extensive and almost unanimous approval which has been given to the plan proposed. If we consider that a large proportion of the scientific men of the country belong to the Medical Profession—that individually they are possessed of the high respect of the communities in which they reside, while as a body their influence upon the interests, welfare and public mind of the nation is almost unfelt, it must be evident that something more is necessary to bestow upon them as a class, the rank to which they are justly entitled. This we have often in our humble sphere stated. It is *Union*, *Association*,—an organization by which our common wants and interests may be made known and protected.

We look to this Convention to effect these objects in whatever way may appear most feasible after due deliberation. Difference of views is of course to be expected, but these, it is believed, may be harmonized, where the objects to be attained and the interests to be favored, do not clash. There is, nevertheless, a single point in the proceedings of the New York State Medical Society, which it was easy to foresee might give rise to objections, and even prove a source of embarrassment to the action of the Convention. We allude to the appointment of delegates from each Senatorial district by the central society, instead of allowing these to be elected by the different county societies. That these latter might in some instances claim the privilege of appointing delegates was to be expected, and we learn from the Buffalo Medical Journal, that such is the case. It is not our object to express an opinion in regard to the wisdom or justice of the course adopted by the State Society, we should greatly regret that anything should arise capable of interfering with the harmonious action of the Convention, but do not at present see reason to apprehend such a result.

We have used every effort to have the profession of this State fully represented. At a late meeting of the Trustees of Rush Medical College, Dr. Brainard was appointed delegate, with power to associate with himself any of his colleagues who might be able to attend, and we have urged upon the Medical associations known to exist in the State, the importance of being also represented. To what extent our recommendations have been, or may be effectual, we are at present unable to say. D. B.

